# Sustained density of neuroendocrine cells with aging precedes development of prostatic hyperplasia in spontaneously hypertensive rats

**DOI:** 10.1186/s12894-019-0528-7

**Published:** 2019-10-16

**Authors:** Yuki Kyoda, Koji Ichihara, Kohei Hashimoto, Ko Kobayashi, Fumimasa Fukuta, Naoya Masumori

**Affiliations:** Department of Urology, Sapporo Medical University School of Medicine, S. 1, W. 16, Chuo-ku, Sapporo, 060-8543 Japan

**Keywords:** Neuroendocrine cell, Prostatic hyperplasia, Spontaneously hypertensive rat

## Abstract

**Background:**

Neuroendocrine (NE) cells may have an impact on the development and initial growth of benign prostatic hyperplasia (BPH) according to previous human studies.

**Methods:**

To explore the relationship of NE cells and BPH development, we compared the density of NE cells and also prostatic weight in spontaneously hypertensive rats (SHR), which develop by aging, and Wistar-Kyoto rats (WKY) as control. The total weights of the epithelium and stroma in the ventral lobes of 8-, 12, 16-, 28- and 56-week-old SHR and WKY were calculated using Image J software. NE cells in the ventral prostatic ducts (VPd) were quantified using immunohistochemical staining for serotonin.

**Results:**

Although there was no significant difference in the estimated total weight of the epithelium and stroma in the ventral lobes adjusted by body weight (ES weight) between the two groups at 8, 12 and 16 weeks of age, ES weight was significantly greater in the SHR group than in the WKT group at 28 and 56 weeks. The density of NE cells in the VPd decreased with aging in the WKY group, whereas it was sustained until 16 weeks and then decreased with aging in the SHR group. The difference in the density between the two groups was most marked at 16 weeks of age.

**Conclusion:**

In the natural history of BPH, NE cells may play an important role in the initial development of BPH because sustained density of NE cells in the VPd precedes the development of prostatic hyperplasia.

## Background

Benign prostatic hyperplasia (BPH) is a common disease that causes lower urinary tract symptoms in elderly men. However, we do not precisely know its etiology, although chronic inflammation, the change of hormonal status and aging have an influence on it [1–3].

Neuroendocrine (NE) cells are one of epithelial cells of the prostate. Their function is to maintain homeostasis in the prostate by producing a variety of neurosecretory products having growth-promoting activities, including serotonin (5-HT), calcitonin, parathyroid hormone-related peptides and so on [4, 5]. In addition, NE cells are involved in carcinogenesis and progression of prostate cancer as well as development of castration resistance prostate cancer [6–8]. On the other hand, few studies have examined the relationship between NE cells and human BPH [9–11]. Cockett et al. reported that there were many NE cells in and around small adenomatous nodules, which are the main pathological finding of BPH, whereas there are few NE cells in large adenomatous nodules [9]. Furthermore, we retrospectively quantified the density of NE cells in the transition zone (TZ) of the human prostate, where adenomatous nodules mainly appear [10]. Since the density of NE cells in the TZ containing small adenomas with normal epithelium and stroma around them in the TZ was higher than in the TZ containing large adenoma or no adenoma, we hypothesized that NE cells might have an influence on the initial growth of BPH.

The epithelium of the rat prostate is different from that of the human prostate because the rat epithelium lacks NE cells [12]. NE cells in the Sprague-Dawley rats are distributed only in the urethra and the periurethral ducts, which link the urethra and the prostatic lobes, but not in the prostatic lobes. The density of NE cells in the periurethral ducts of the Wistar rat peaks in puberty and then decreases as the rats become older [13]. It is known that Sprague-Dawley and Wistar rats have no findings of prostatic hyperplasia with aging. On the other hand, no studies have reported on NE cells in the periurethral ducts of the spontaneously hypertensive rat (SHR), which is a model of BPH. SHR have been widely studied as an experimental model of hypertension and revealed their features of benign adenomatous hyperplasia in the ventral prostate of old SHR [14]. Previous studies demonstrated hyperplastic changes in the ventral prostate from young to older SHR, and close associations between the degree of its hyperplasia and aging in SHR [14–16]. The development of prostatic hyperplasia may result from both epithelial and stromal growth in SHR with age and may be predominantly expressed as a glandular type in SHR [16]. Thus, we compared the density of NE cells of the Wistar Kyoto rat (WKY), normotensive counterparts of SHR, to that of the SHR to investigate the relationship between NE cells and prostatic hyperplasia. Moreover, since few studies have reported when the hyperplasia begins in the prostate of SHR, we histologically evaluated the time of the initial development of prostatic hyperplasia by using image analysis software.

## Methods

All surgical procedures and experimental protocols complied with institutional guidelines and were reviewed and approved by the Animal Care and Use Committee of Sapporo Medical University (#15–026).

### Animals and experimental procedure

In this study, 8-, 12-, 16-, 28- and 56-week-old male SHR and WKY rats purchased from CHARLES RIVER LABORATORIES JAPAN, INC. (Yokohama, Japan) were used. They were bred in specific pathogen free room. The number of rats analyzed for each age group was five for both the SHR and WKY groups. Their body weights were measured and they were euthanized under isoflurane (2–4%) anesthesia by cervical dislocation. Thereafter, an abdominal incision was made to identify the abdominal cavity and they underwent cystoprostatectomy with simultaneous partial urethrectomy by cutting of the urethra axially at the level of pubic height. Each specimen was microscopically divided into 7 parts: 2 ventral lobes, 2 dorsolateral lobes, 2 coagulating glands, an ampullary gland, 2 seminal vesicles, the bladder and part of the urethra in saline. The weight of bilateral ventral lobes was measured using an electronic analytical scale.

### Histopathological examination

**Fig. 1:**
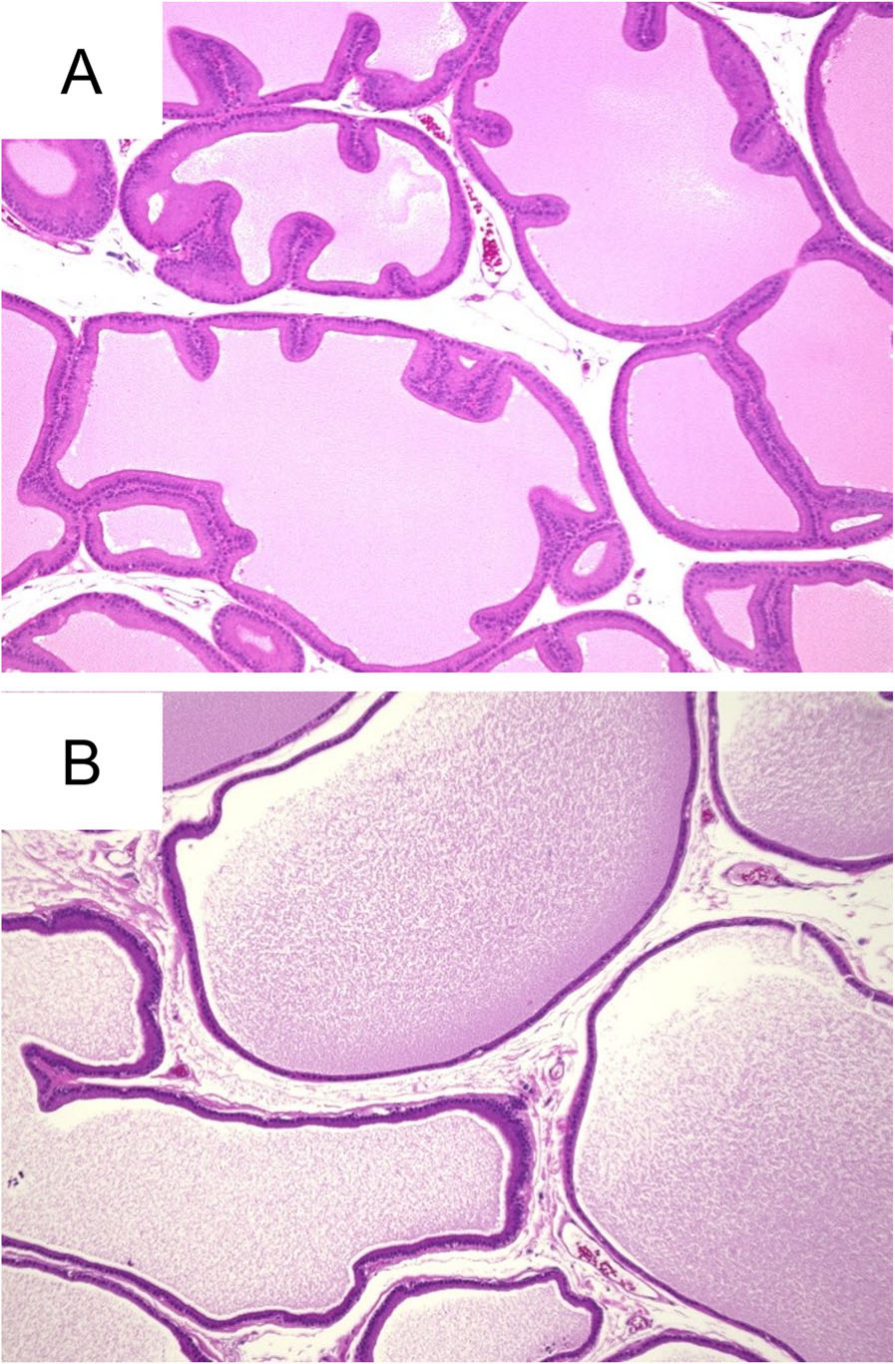
Observation of epithelia and stromal cells in ventral prostate using hematoxylin and eosin staining. **a**: 56-week-old SHR, Spontaneously hypertensive rat; **b**: 56-week-old WKY, Wistar Kyoto rat. (× 200)

The divided specimens were fixed in 15% neutral-buffered formalin (Wako Pure Chemical Industries, Ltd., Osaka, Japan) for 48–96 h. The specimens were then embedded in paraffin, cut into 5-μm sections and stained with hematoxylin and eosin (Fig. 1) [10].

Immunohistochemical staining was carried out using polyclonal antibodies for serotonin. It was done on 4-μm-thick paraffin sections cut from the formalin-fixed tissues. Heat-induced epitope retrieval was performed in Tris-EDTA buffer (10 mM Tris Base, 1 mM EDTA solution, 0.05% Tween 20, pH 9.0). The sections were then incubated in 3% H2O2 for 10 min to eliminate endogenous peroxidase activity. The primary rabbit anti-serotonin polyclonal antibody (1,20,000 Immunostar, Stillwater, MN) and a horseradish peroxidase-conjugated anti-rabbit secondary antibody were used at room temperature for 60 min and 30 min, respectively [10]. Color development was accomplished with 3,3′-diaminobenzidine. The nuclei were then counterstained with hematoxylin. Only manifest cytoplasmic staining was defined as a positive reaction. Negative controls were incubated with normal rabbit serum instead of the primary antibody [10].

### Histological analysis of hyperplasia of the prostate

**Fig. 2:**
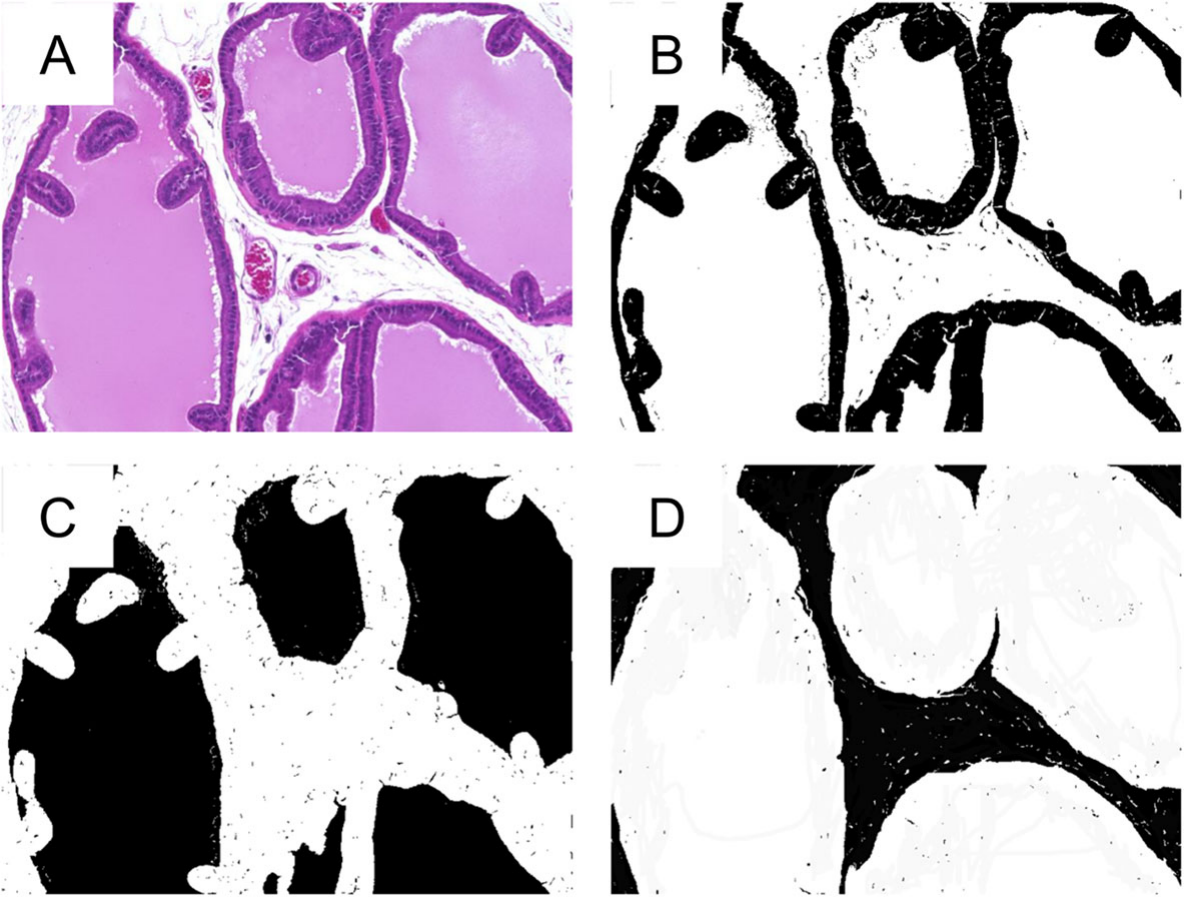
Histological evaluation of the ventral lobe of the rat prostate. Percent areas of epithelium and stroma, lumen, and matrix in 5 randomly selected areas from one of bilateral ventral lobes were calculated using Image J software. **a**: Hematoxylin and eosin stain; **b**: Black area, epithelium and stroma; **c**: Black area, lumen; **d**: Black area, matrix. (× 200)

Rats have much more lumen and matrix in the prostate than humans. Thus, the whole weight of the rat ventral lobes does not reflect hyperplasia histologically. Five randomly selected areas from one of bilateral ventral lobes captured by microscopy under 200-fold magnification. The percent areas of epithelium and stroma, the lumen, and matrix were calculated using Image J software (Rasband, W.S., ImageJ, U. S. NIH, Bethesda, Maryland) (Fig. 2). We estimated the total weight of the epithelium and stroma in the ventral lobe adjusted by body weight (ES weight) as follow; (percent area of epithelium and stroma x weight of bilateral ventral lobes)/body weight of rat (mg/g).

### Analysis of density of NE cells

**Fig. 3:**
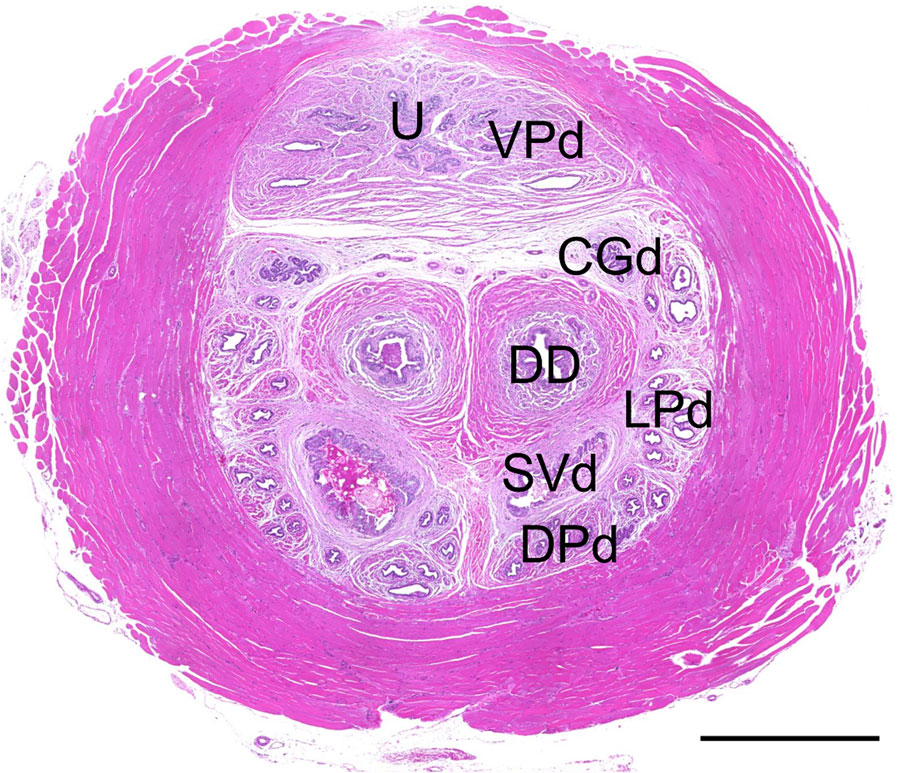
Cross section of proximal urethra consisting of the periurethral ducts around the urethra. CGd, coagulating gland duct; DD, deferent duct; DPd, dorsal prostatic duct; LPd, lateral prostatic duct; SVd, seminal vesicle duct; U, urethra; VPd, ventral prostatic duct. Scale bar: 1 mm

We evaluated the density of NE cells in the epithelia of the ventral prostatic ducts (VPd) in a cross section of the urethra. This section, which was 1.5 mm from the bladder contained a urethra, 10 to 16 ventral prostatic ducts (VPd), 32 to 40 dorsolateral prostatic ducts (DLPd), 2 coagulating gland ducts (CGd), 2 seminal vesicle ducts (SVd) and 2 deferent ducts (DD) (Fig. 3). NE cells were distributed in the epithelia of the periurethral ducts and urethra. The density of NE cells in the VPd was calculated as the ratio of serotonin-positive NE cells among the total number of epithelial cells, which was manually counted in high power fields of a microscope (BZ-9000, KEYENCE, Tokyo, Japan).

### Statistical analysis

Statistical comparisons were made using the unpaired t-test and Mann-Whitney U-test. *P* < 0.05 was considered to be statistically significant. These analyses were performed using SPSS®, v. 23.0 (SPSS Inc., Chicago, IL).

## Results

The weights of the ventral lobes in SHR were significantly lower than in WKY except in rats 28 weeks old, although their body weights were nearly the same (Table 1).

**Table 1:** Body weights and weights of ventral lobes in rat

Weight	SHR^a^ (*n* = 5)	WKY^b^ (*n* = 5)	*P* value
Mean body weight (g) ± S.E.			
8 weeks old	228 ± 9.7	216 ± 5.1	0.521
12 weeks old	314 ± 11.7	298 ± 12.8	0.823
16 weeks old	338 ± 6.6	338 ± 5.8	> 0.999
28 weeks old	378 ± 13.5	396 ± 7.5	0.344
56 weeks old	444 ± 13.3	456 ± 6.9	0.340
Bilateral ventral lobes (mg) ± S.E.			
8 weeks old	203 ± 18.8	303 ± 15.6	0.021
12 weeks old	384 ± 15.5	560 ± 40.0	0.016
16 weeks old	425 ± 10.4	580 ± 38.4	0.008
28 weeks old	595 ± 44.7	772 ± 40.7	0.056
56 weeks old	585 ± 51.7	832 ± 62.2	0.032

^a^Spontaneously hypertensive rat

^b^Wistar Kyoto rat

### Evaluation of estimated total weight of epithelium and stroma in ventral lobe of the prostate

Image J quantified the areas of the epithelium and stroma, lumen, and matrix in the prostatic ventral lobes of SHR and WKY (Table 2). The percent area of epithelium and stroma in SHR was significantly larger than that of WKY for each age week analyzed. In WKY, the percent area of epithelium and stroma were less than that of the lumen and matrix for all ages.

**Table 2:** Percent areas of epithelium and stroma, lumen, and matrix of ventral lobes in rat

Percent area (mean ± S.E.)	SHR^a^ (*n* = 5)	WKY^b^ (*n* = 5)	*P* value
Epithelium and stroma			
8 weeks old	42.7 ± 3.1	28.8 ± 1.9	0.016
12 weeks old	39.5 ± 2.1	24.0 ± 2.1	0.008
16 weeks old	36.3 ± 1.1	24.7 ± 2.6	0.008
28 weeks old	33.2 ± 4.6	21.8 ± 3.4	0.008
56 weeks old	41.8 ± 5.6	22.4 ± 2.5	0.016
Lumen			
8 weeks old	32.3 ± 3.4	41.6 ± 1.6	0.056
12 weeks old	39.1 ± 2.1	48.6 ± 2.3	0.016
16 weeks old	37.1 ± 0.8	48.4 ± 4.6	0.151
28 weeks old	43.0 ± 4.3	51.3 ± 3.8	0.032
56 weeks old	39.1 ± 4.2	44.2 ± 4.1	0.421
Matrix			
8 weeks old	25.0 ± 3.2	29.6 ± 2.0	0.222
12 weeks old	21.3 ± 2.2	27.4 ± 1.8	0.056
16 weeks old	26.6 ± 1.0	26.9 ± 4.1	0.690
28 weeks old	23.8 ± 2.0	26.9 ± 1.7	0.548
56 weeks old	19.1 ± 2.9	33.4 ± 2.9	0.016

^a^Spontaneously hypertensive rat

^b^Wistar Kyoto rat

**Fig. 4:**
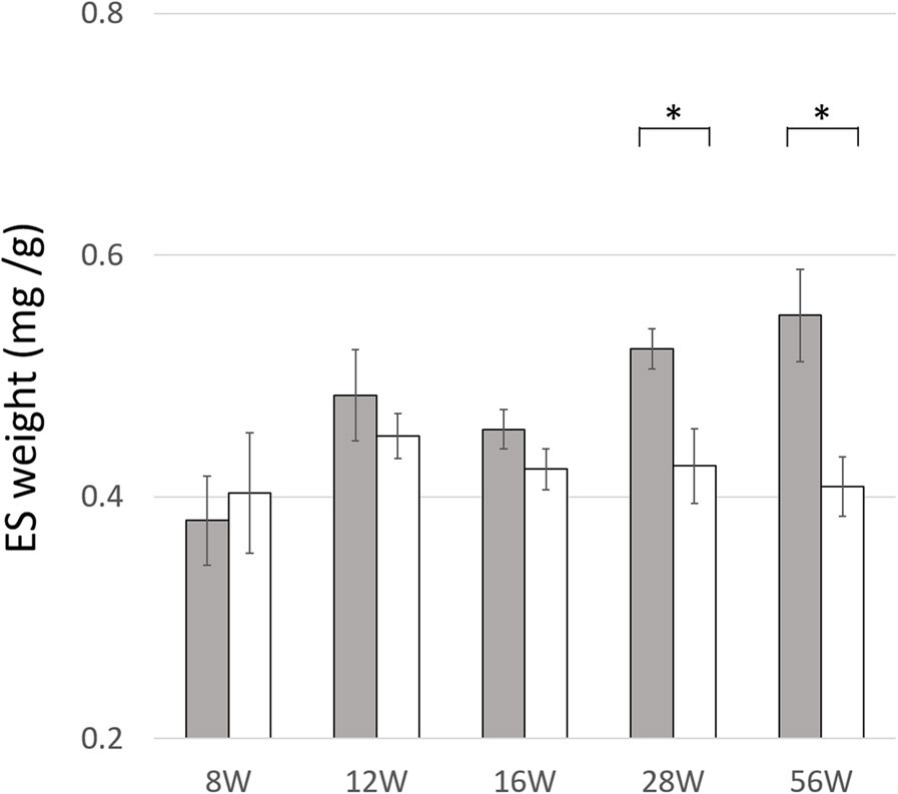
Estimated total weight of epithelium and stroma in the ventral lobe per body in rats. Grey columns, SHR; white columns, WKY. The estimated total weights of the epithelium and stroma in the ventral lobe per body weight (mean ± S.E.) in SHR vs. WKY were 0.38 ± 0.04 vs. 0.40 ± 0.05 in 8-week old rats (*p* = 0.624), 0.48 ± 0.04 vs. 0.45 ± 0.02 in 12-week old rats (*p* = 0.303), 0.46 ± 0.02 vs. 0.42 ± 0.02 in 16-week old rats (*p* = 0.090), 0.52 ± 0.02 vs. 0.43 ± 0.03 in 28-week old rats (*p* = 0.03) and 0.55 ± 0.04 vs. 0.41 ± 0.02 in 56-week-old rats (*p* = 0.014). Significant differences in weight were observed between SHR and WKY at 28 and 56 weeks of age

The ES weights are shown in Fig. 4. Although there were no differences in the ES weights between SHR and WKY at 8, 12 and 16 weeks of age, significant differences were observed at 28 and 56 weeks of age (*p* = 0.034 and *p* = 0.014).

### Density of NE cells in the ventral prostatic ducts

**Fig. 5:**
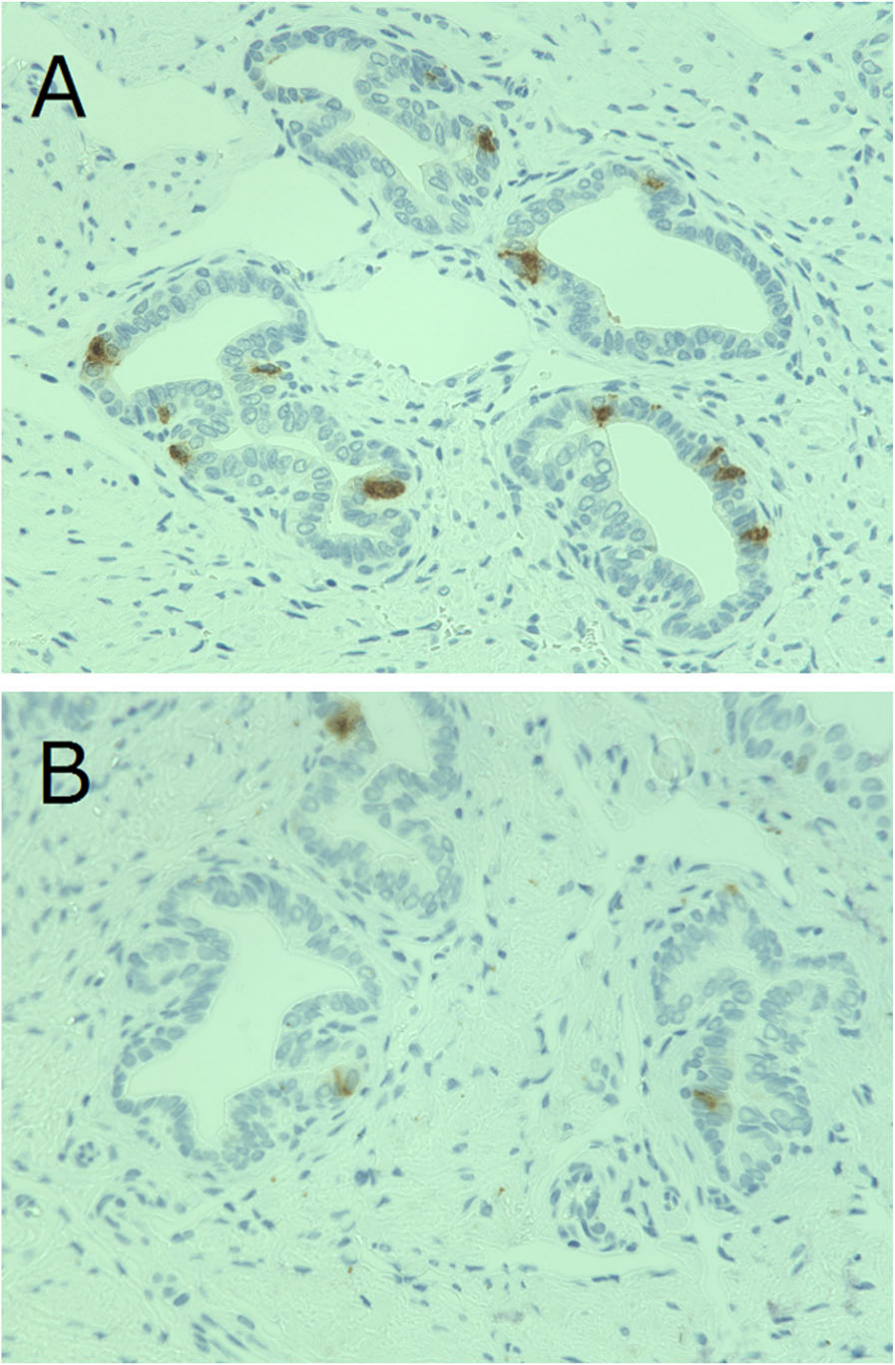
Immunohistochemical staining of serotonin in the ventral prostatic ducts. Ventral prostatic ducts of (**a**): 16-week-old SHR and (**b**): 16-week-old WKY. (× 400)

**Fig. 6:**
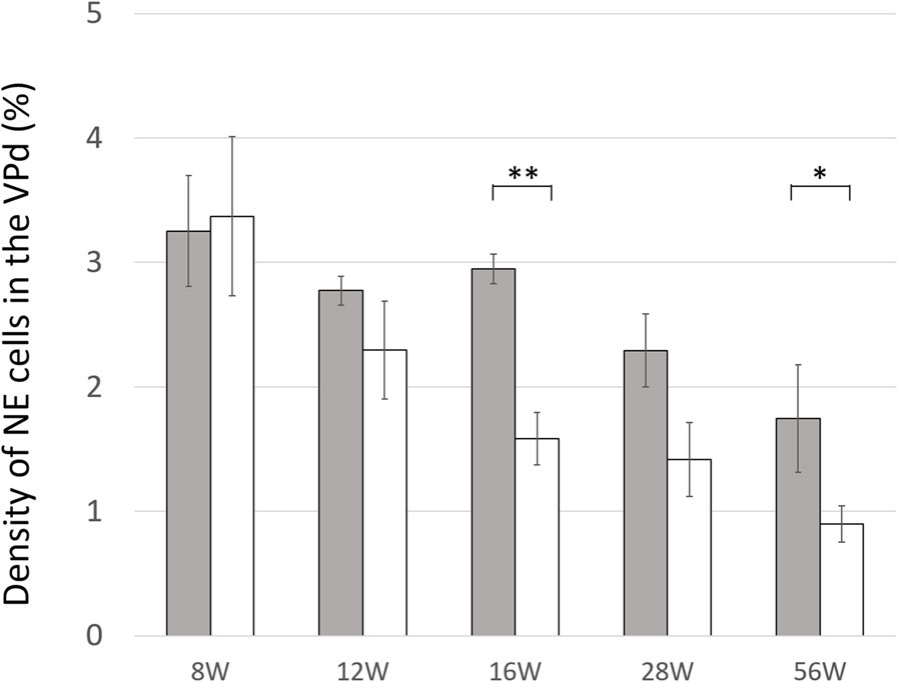
Density of NE cells in the epithelium of ventral prostatic ducts. Grey columns, SHR; white columns, WKY. The density of serotonin-positive NE cells to all epithelial cells (mean ± SE) (%) in SHR vs. WKY were 3.25 ± 0.44 vs. 3.37 ± 0.64 in 8-week old rats (*p* = 0.882), 2.77 ± 0.12 vs. 2.30 ± 0.39 in 12-week old rats (*p* = 0.117), 2.95 ± 0.12 vs. 1.58 ± 0.21 in 16-week old rats (*p* < 0.001), 2.29 ± 0.29 vs. 1.42 ± 0.30 in 28-week old rats (*p* = 0.069) and 1.75 ± 0.43 vs. 0.90 ± 0.14 in 56-week old rats (*p* = 0.043). The density of NE cells in the VPd decreased with aging in the WKY group, whereas it was sustained by 16 weeks old, then decreased with aging in the SHR group. There was the most remarkable difference in the density between the two groups at 16 weeks old (*p* < 0.001)

Figure 5 shows immunohistochemical staining of serotonin in the VPd. The density of NE cells in the VPd decreased with aging in the WKY group, whereas it was sustained until 16 weeks of age and then decreased with aging in the SHR group (Fig. 6). The difference in the density between the two groups was greatest at 16 weeks of age.

## Discussion

Our previous study using human prostates demonstrated that NE cells were plentiful in the TZ having a small adenomatous nodule compared to the TZ having no adenomatous nodule or a large adenomatous nodule [10]. Thus, we hypothesized that NE cells had an impact on the initial growth of BPH in a paracrine fashion. In the present study using animal models, we found that the density of NE cells in the VPd was much larger in 16-week-old SHR than in WKY rats. After the age of 16 weeks, the difference in the ES weight in the ventral lobes of SHR and WKY became remarkable. Thus, sustained density of NE cells in the VPd preceded development of prostatic hyperplasia. The main factors initiating BPH are not known, although many factors such as chronic inflammation, for instance, which influences the growth of adenoma in the progressive period, have been reported [1]. NE cells may influence the initial growth of prostatic hyperplasia as one of the initiators, although they have negligible or negative impact on it in the progressive period. Carvalho-Dias et al. reported that 5-HT, one of the products by NE cells is decreased in BPH through modulation of androgen receptor [17].

Based on the ES weights in the ventral lobes, the present study showed a progressive degree of prostatic hyperplasia in SHR. The rat prostate contains more fluid in the lumen than the human one, so it is incorrect to consider that the whole weight of the ventral lobes reflects the degree of prostatic hyperplasia because it is easily influenced by fluid weight rather than actual histological weight. In fact, the mean weight of the bilateral ventral lobes in SHR had a tendency to be lower than that of the WKY rat even when the SHR histologically had hyperplasia in the ventral lobes. On the other hand, the ES weight estimated by adjustment of the percent area of the epithelium and stroma, as well as the body weight of the rat, was greater in SHR than in WKY at 28 weeks and older. Although a few reports have examined prostatic hyperplasia in SHR, no report has clarified the beginning of the histological progression of the hyperplasia because they did not examine the histology before 15 weeks of age [16, 18]. We considered that the difference of the ES weight in the ventral lobes between SHR and WKY rats represented the histological progression of hyperplasia in the prostate with aging.

Rats have no NE cells in the ventral lobe, which corresponds to the TZ of the human prostate [12]. NE cells that are present in the periurethral duct work in a paracrine fashion and may have an effect on the epithelial and stromal cells of the ventral lobe via the duct. In the VPd, there are many open-type NE cells that release serotonin into the lumen [12]. Closed-type NE cells, on the other hand, release it into the inter- and subepithelial spaces. The presence of NE cells is restricted to the terminal (periurethral) portion of the tributary ducts of the lateral and ventral lobes, which are located in the initial segment of the pelvic urethra [8]. Therefore, we evaluated the periurethral duct in the portion where VPds join the urethra. Furthermore, it would be appropriate to evaluate the NE cells in the VPd, because NE cells are more frequent in the periurethral ducts than in the peripheral parts of the gland in the human prostate [9].

There are several limitations in the present study. Firstly, we adopted the SHR as a BPH model. Though this is a common model it has a serious drawback in that the SHR predominantly has epithelium in the prostate compared to stroma in the human prostate and the prostate slightly enlarges. However, we have no alternative ideal animal model of BPH. Second, we only used serotonin as an immunohistochemical marker to evaluate NE cells. It is impossible to detect all NE cells in the prostate even though multiple NE markers are used because, of the more than 200 secretory products, there is no universal one that every NE cell secretes [5]. In the present study, we selected serotonin as a representative NE marker according to previous reports using rats [12]. Finally, a critical limitation of the present study is that we do not know how prostatic NE cells work for the development of prostatic hyperplasia, because we could not demonstrate direct evidence to support the NE cells in the VPd promoted the growth of the ventral lobes which are located above the VPd. However, we believe the present study, which demonstrates that sustained density of NE cells in the VPd precedes the development of prostatic hyperplasia in rats, provides a clue suggesting that NE cells have an impact on the initial growth of BPH as we hypothesized in our previous human study [10].

## Conclusions

NE cells may have an impact on the initial growth of BPH. However, further studies are needed to clarify their function in BPH.

## Data Availability

The datasets used and/or analysed during the current study available from the corresponding author on reasonable request.

## Abbreviations

BPH: Benign prostatic hyperplasia
CGd: Coagulating gland ducts
DD: Deferent ducts
DLPd: Dorsolateral prostatic ducts
ES weight: Estimated total weight of the epithelium and stroma in the ventral lobes adjusted by body weight
NE: Neuroendocrine
SHR: Spontaneously hypertensive rats
SVd: Seminal vesicle ducts
TZ: Transition zone
VPd: Ventral prostatic ducts
WKY: Wistar-Kyoto rats
